# Venous Sinus Stenting for Idiopathic Intracranial Hypertension: A Report of Two Cases

**DOI:** 10.7759/cureus.71326

**Published:** 2024-10-12

**Authors:** Yohei Takenobu, Tao Yang, Noriko Nomura, Manabu Inoue, Kenji Hashimoto

**Affiliations:** 1 Department of Neurology, Osaka Red Cross Hospital, Osaka, JPN; 2 Department of Neurosurgery, Osaka Red Cross Hospital, Osaka, JPN

**Keywords:** idiopathic intracranial hypertension, vein of labbé, venous sinus stenosis, venous sinus stenting, visual impairment

## Abstract

Idiopathic intracranial hypertension (IIH) is characterized by an elevated intracranial pressure of unknown cause, which can lead to severe and sometimes irreversible visual impairment. Recently, venous sinus stenting (VSS) has emerged as an alternative treatment option for IIH. Here, we report two patients with IIH who successfully underwent VSS in the transverse sinus and displayed rapid improvement in visual symptoms. Two young women presented with progressive visual symptoms of papilledema. The cerebrospinal fluid (CSF) opening pressures were elevated higher than 40 cmH_2_O. Magnetic resonance venography demonstrated stenosis of the transverse sinuses. Intravascular ultrasonography demonstrated focal extrinsic narrowing of the sinuses. Dilatation of the stenotic sinuses using self-expanding open-cell stents resolved the trans-stenotic pressure gradients and lowered the CSF pressure. Visual symptoms and papilledema improved immediately after the procedures. VSS could be a useful treatment option for IIH with rapidly progressive visual impairment.

## Introduction

Idiopathic intracranial hypertension (IIH) is a rare neurological disorder characterized by elevated intracranial pressure of unknown cause, which can lead to significant morbidity, including severe and sometimes irreversible visual impairment, and debilitating headaches [1,2]. Both medical and surgical interventions have been developed for its treatment. Recently, venous sinus stenting (VSS) has emerged as a potential therapeutic option to address the underlying venous outflow abnormalities associated with IIH [3-7]. In this report, we describe two cases of IIH with progressive visual impairment and stenosis of the transverse sinus, in which VSS successfully reduced cerebrospinal fluid (CSF) pressure and improved visual symptoms.

## Case presentation

Case 1

**Figure 1:**
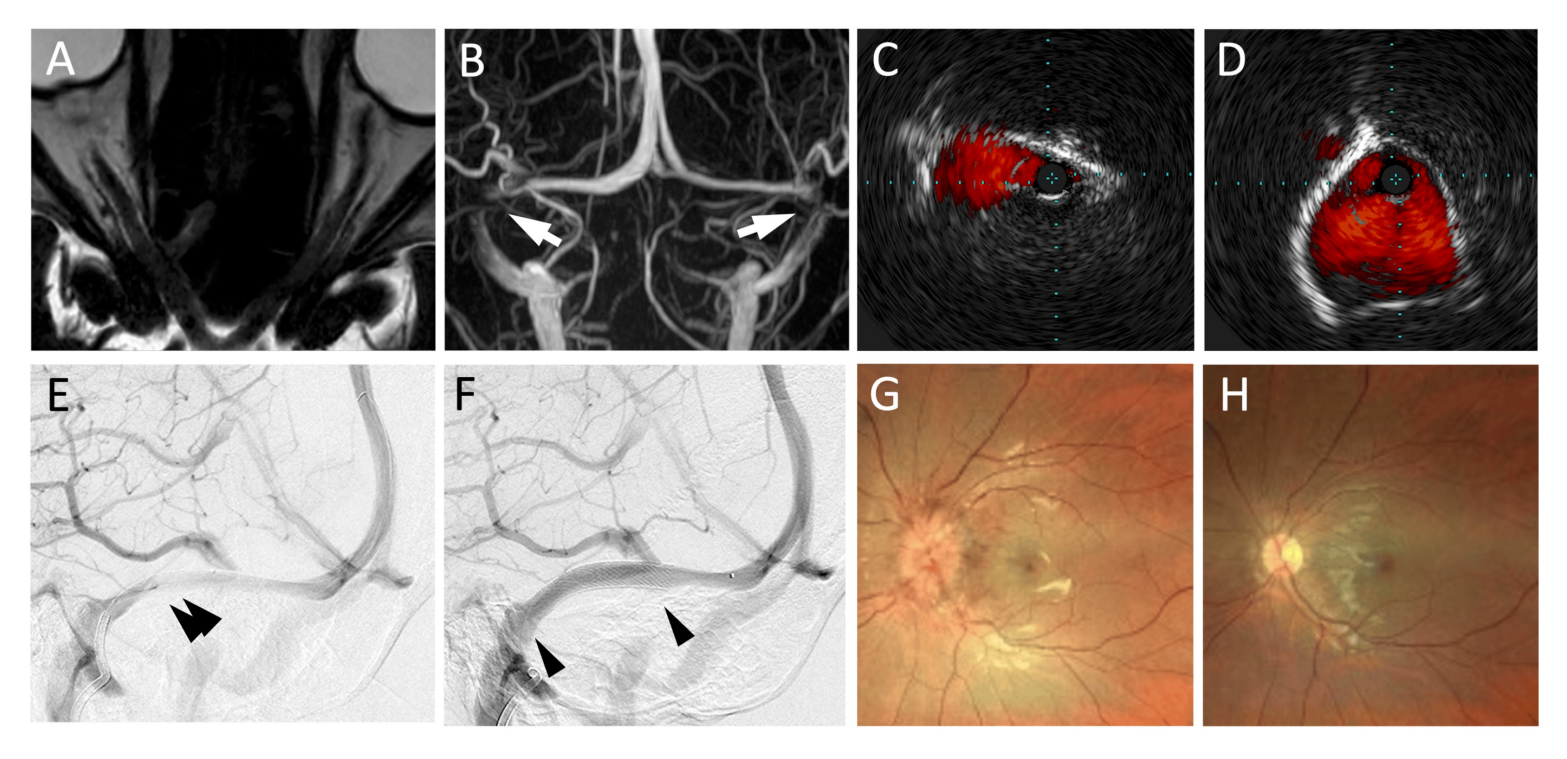
Images from Case 1 (A) MRI on admission revealed optic nerve tortuosity, enlargement of the peripapillary subarachnoid space, and flattening of the dorsal sclera of the eyes. (B) MRI venography showing bilateral stenosis of the transverse sinuses (arrow). (C) IVUS image shows the triangular extrinsic stenosis at the transverse-sigmoid sinus junction. (D) IVUS image provides precise measurements of the medial part of the transverse sinus, helping determine the stent size. (The distance between blue dots represents 1 mm.) (E) Right internal cerebral angiogram demonstrated transverse sinus stenosis (double arrow). (F) Angiogram after stenting exhibited the dilatation of the transverse sinus. The stent (between the two arrowheads) did not disturb the flow in the vein of Labbé, although it covered the vein’s orifice. (G)-(H) Ophthalmoscopic examination revealed severe papilledema on admission (G) and its recovery on postoperative day 7 (H). MRI: magnetic resonance imaging, IVUS: intravascular ultrasonography.

A 26-year-old woman, height of 158 cm, weighing 118 kg, and body mass index of 47.3 kg/m^2^, presented to an ophthalmology clinic with progressive visual field disturbance two weeks before. She was referred to our hospital for evaluation of papilledema. Magnetic resonance imaging (MRI) demonstrated optic nerve tortuosity, peripapillary subarachnoid space enlargement, and dorsal sclera flattening of the eyes (Figure 1A). The bilateral transverse sinuses exhibited stenosis on MRI venography (Figure 1B). As the lumbar puncture revealed CSF pressure higher than 50 cmH_2_O and no underlying disease was found, a diagnosis of IIH was made. Diagnostic angiography with pressure measurements revealed a trans-stenotic pressure gradient of 9 mmHg. The patient did not respond to oral isosorbide, and progressive visual symptoms required immediate lowering of the CSF pressure. We planned VSS because the malfunction of the CSF diversion was expected due to the dense subcutaneous tissue. Dual antiplatelet therapy with aspirin and clopidogrel was administered seven days before the procedure. Under local anesthesia, a diagnostic catheter was placed in the right internal carotid artery from the femoral artery, and an 8-Fr guiding catheter was placed from the femoral vein into the right internal jugular vein. Intravascular ultrasonography (IVUS) for the dural sinus demonstrated extrinsic triangular narrowing without intraluminal components (Figure 1C). After IVUS measuring of the normal sinus diameter (Figure 1D), a 7 × 40 mm Precise Pro Rx (Cordis; Miami Lakes, FL, USA) was deployed. Although the stent covered the opening of the vein of Labbé (VOL), located in the middle of the transverse sinus, the VOL flow remained antegrade (Figures 1E, 1F). Immediately after stenting, the trans-stenotic pressure gradient decreased to 1 mmHg, and the CSF pressure decreased to 38 cmH_2_O. After one week, the visual field deficits and papilledema improved (Figures 1G, 1H). Fourteen months after the procedure, the visual function was well maintained. Computed tomography (CT) venography revealed no filling defects in the stent. Aspirin alone was administered to the patient.

Case 2

**Figure 2:**
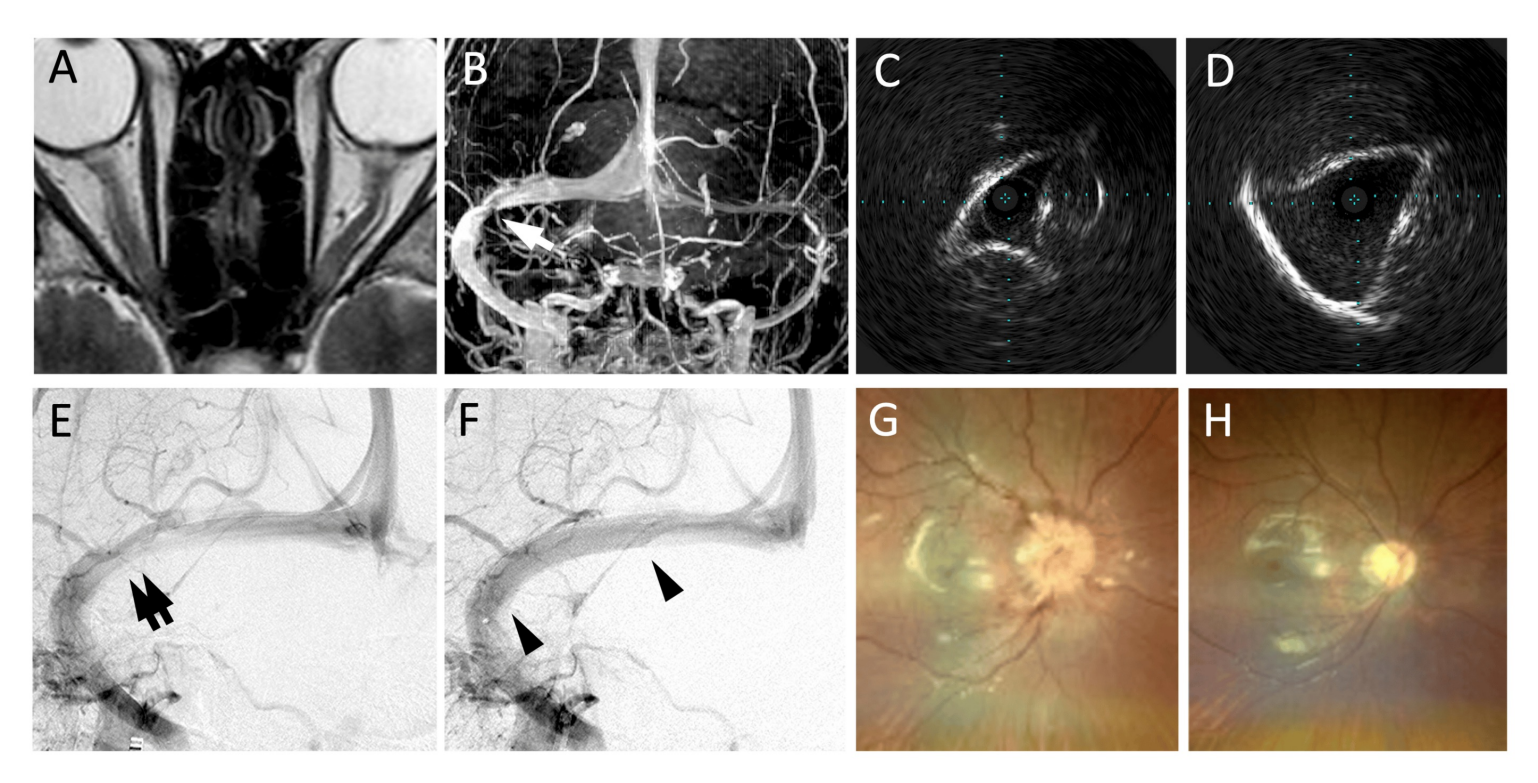
Images from Case 2 (A) MRI on admission displayed optic nerve tortuosity, enlargement of the peripapillary subarachnoid space, and flattening of the dorsal sclera of the eyes. (B) Contrast-enhanced MRI venography demonstrated stenosis of the right transverse sinus (arrow), whereas the left transverse-sigmoid sinus was hypoplastic. (C) IVUS image shows the triangular extrinsic stenosis at the transverse-sigmoid junction. (D) IVUS image provides precise measurements of the medial part of the transverse sinus, helping determine the stent size. (The distance between blue dots represents 1 mm.) (E) Right internal cerebral angiogram demonstrated stenosis of the transverse sinus (double arrow). (F) Angiogram after stenting exhibited the dilatation of the transverse sinus. The stent (between the two arrowheads) did not disturb the flow of the vein of Labbé. (G)-(H) Ophthalmoscopic examination revealed severe papilledema on admission (G) and its recovery at postoperative day 20 (H). MRI: magnetic resonance imaging, IVUS: intravascular ultrasonography.

A 27-year-old woman, height of 162 cm, weighing 115 kg, and body mass index of 43.8 kg/m^2^, presented to our hospital via the ophthalmology department for progressive vision loss and visual field disturbance three weeks before. Her visual acuity on the left side had decreased to 20/500. Mariott’s blind spots were enlarged bilaterally, and a central scotoma appeared on the left side. MRI demonstrated optic nerve tortuosity, peripapillary subarachnoid space enlargement, and dorsal sclera flattening of the eyes (Figure 2A). Lumbar puncture revealed CSF pressure of 40 cmH_2_O. MRI venography revealed hypoplasia of the left transverse-sigmoid sinus and stenosis of the right transverse sinus (Figure 2B). Diagnostic angiography with pressure measurements revealed a trans-stenotic pressure gradient of 19 mmHg. As the patient did not respond to oral acetazolamide and her vision progressively deteriorated, VSS was planned. Dual antiplatelet therapy with aspirin and prasugrel was administered seven days before the procedure. Under local anesthesia, a diagnostic catheter was placed in the right internal carotid artery from the right radial artery, and an 8-Fr guiding catheter was inserted into the internal jugular vein from the right femoral vein. IVUS demonstrated extrinsic triangular narrowing of the transverse sinus without intraluminal components (Figure 2C). After measuring of the normal sinus diameter using IVUS (Figure 2D), a 7 × 40 mm Precise Pro Rx was deployed. Although the stent covered the opening of the VOL, the VOL flow remained antegrade (Figures 2E, 2F). The pressure gradient decreased to 0 mmHg, and the CSF pressure decreased to 27 cmH_2_O. The visual acuity of the left eye recovered to 20/100 in one week, and the visual field deficits and papilledema improved (Figures 2G, 2H). Twelve months after the procedure, the visual symptoms improved, and CT venography displayed no filling defects in the stent.

## Discussion

IIH, formerly known as pseudotumor cerebri, is a rare neurologic disorder characterized by increased intracranial pressure without an identifiable cause, largely affecting overweight (body mass index ≥ 30 kg/m^2^) women of reproductive age [1,2,8]. IIH poses diagnostic challenges due to a lack of specific markers. The diagnostic criteria for typical IIH consist of elevated intracranial pressure (≥ 25 cmH_2_O) presenting papilledema, without hydrocephalus or space-occupying lesions, normal CSF composition, and without no underlying etiology such as endocrine disorder or venous sinus thrombosis [2,9]. A certain degree of visual impairment is present in 95% of the cases, and permanent visual impairment and blindness occur up to 25% and 10%, respectively [1], requiring early detection and treatment. Our patients demonstrated progressive visual symptoms and elevated CSF pressure (case 1: 50 cmH_2_O and case 2: 40 cmH_2_O), and no other disease-causing intracranial hypertension was found on imaging, blood, or CSF tests. Therefore, fulminant IIH associated with obesity was diagnosed.

Recently, MRI has enabled the detection of secondary changes including elevated intracranial pressure, such as an enlarged peripapillary subarachnoid space, optic nerve tortuosity, flattening of the dorsal sclera of the eyes, an enlarged sella turcica, and stenosis of the transverse sinus [1,8,9]. Sinus stenosis of 50% or greater is observed in 71% of patients with IIH [10]. Whether dural sinus stenosis is a primary cause or a secondary consequence of IIH is still under debate. Regardless of the cause, dural sinus stenosis can obstruct venous drainage, further increasing the intracranial pressure and worsening sinus collapse. Breaking this pathological cycle is necessary to treat IIH.

Medical therapy for mild-to-moderate IIH is often successful with acetazolamide [11] or topiramate [12], which potentially modifies the production and absorption of CSF. The consensus guidelines published in 2018 state that surgical treatment such as CSF diversion with a ventriculo-peritoneum or lumbar-peritoneum shunt or optic nerve sheath fenestration (ONSF) should be considered in the case of progressive vision loss [8]. These surgical approaches can immediately lower the intracranial pressure. Although the effectiveness of CSF diversion is well established, this intervention is associated with a high rate of failure and complications, with revision required in 35% of individuals within the first year [13]. ONSF might be an effective intervention for preserving vision in medically refractory IIH. However, a significant proportion (16.9%) of individuals required supplementary interventions, such as shunting or stenting, to achieve symptom control despite initially successful ONSF. Although the efficacy of VSS is described as “not established” in the 2018 consensus guidelines [8], with the recent development of neuroendovascular therapy, IIH patients with venous sinus stenosis and trans-stenotic pressure gradient of 8 mmHg or more are considered candidates for VSS [3-7]. A systematic review including a comparison of surgical approaches for IIH reported that the rates of improved papilledema, severe complications, and treatment failure were 90.5%, 2.2%, and 9.4% in ONSF; 78.9%, 9.4%, and 43.4% in CSF diversion; and 87.1%, 2.3%, and 11.3% in VSS, respectively [13].

As for the technical aspect of VSS, stent designs including open-cell, closed-cell, and dual-layer stents result in favorable outcomes [14,15], whereas Casper-Rx stent (Terumo, Tokyo, Japan) causes easier and more successful delivery with fewer attempts [14]. We selected open-cell carotid stents because the stents had to be placed in the curved segments of the sigmoid sinus, and less metal coverage could reduce the risk of delayed thrombosis. Appropriate positioning of the stent deployment in relation to the confluence of the cortical veins, especially the VOL, must be considered. In VSS involving transverse-sigmoid sinus, drainage of the VOL is affected in 13% to 28.3% of the procedures, depending on stent diameter (≥ 9 mm), although none of them is symptomatic [16,17]. As for post-procedural management, no consensus exists on the antiplatelet therapy regimen and its optimal duration. Dual antiplatelet therapy with aspirin and thienopyridine is a popular combination. Duration of three months and six months or more revealed no significant differences in stent-adjacent or in-stent stenosis in a retrospective study [18]. A randomized controlled trial evaluating CSF shunting versus venous sinus stenting for IIH is being conducted (ISRCTN57142415) to clarify the safety, efficacy, long-term results, optimal regimen, and duration of antithrombotic regimen beyond those retrospective studies.

Our patients had rapidly progressive papilledema and visual impairments and were unresponsive to initial medical therapy. Therefore, an immediate reduction in CSF pressure is required. Because obesity anticipates a considerable degree of shunt malfunction in CSF diversion, the patients underwent VSS. Although the 7-mm, open-cell carotid stents covered the orifices of the VOL, venous drainage did not interfere. After the procedure, CSF pressure immediately decreased, and papilledema and visual impairment immediately improved.

Recently, exenatide, a glucagon-like peptide-1 receptor agonist, and AZD4017, an 11b-hydroxysteroid dehydrogenase type 1, have emerged as candidates for novel medical therapy for IIH [19,20]. Exenatide reduces the intracranial pressure within 24 hours and is expected to have short-term treatment effects. However, phase III trials are required to elucidate the efficacy and safety of those treatments. When patients do not respond to any of the medical treatments and vision loss progresses, immediate reduction with surgery, including dural sinus stenting, is necessary before irreversible vision loss occurs.

## Conclusions

In this report, we described two cases of IIH with dural sinus stenosis who underwent VSS and improved visual function. VSS could be a feasible treatment option for IIH with rapidly progressive and medically refractory visual impairments. Randomized controlled trials comparing VSS with other medical and surgical therapies are warranted.
